# Electron–vibration coupling induced renormalization in the photoemission spectrum of diamondoids

**DOI:** 10.1038/ncomms11327

**Published:** 2016-04-22

**Authors:** Adam Gali, Tamás Demján, Márton Vörös, Gergő Thiering, Elena Cannuccia, Andrea Marini

**Affiliations:** 1 Institute for Solid State Physics and Optics, Wigner Research Centre for Physics, Hungarian Academy of Sciences, PO Box 49, H-1525 Budapest, Hungary; 2 Department of Atomic Physics, Budapest University of Technology and Economics, Budafoki út 8, H-1111 Budapest, Hungary; 3 Institute of Physics, Loránd Eötvös University, Pázmány Péter sétány 1/A, H-1117 Budapest, Hungary; 4 Materials Science Division, Argonne National Laboratory, Lemont, Illinois 60439, USA; 5 Institute for Molecular Engineering, University of Chicago, Chicago, Illinois 60637, USA; 6 Aix-Marseille Université, CNRS, PIIM UMR 7345, 13397 Marseille, France; 7 Institute for Material Science (ISM) of the National Research Council (CNR), Via Salaria Km 29.3, CP 10, 00016 Monterotondo Stazione, Italy

## Abstract

The development of theories and methods devoted to the accurate calculation of the electronic quasi-particle states and levels of molecules, clusters and solids is of prime importance to interpret the experimental data. These quantum systems are often modelled by using the Born–Oppenheimer approximation where the coupling between the electrons and vibrational modes is not fully taken into account, and the electrons are treated as pure quasi-particles. Here, we show that in small diamond cages, called diamondoids, the electron–vibration coupling leads to the breakdown of the electron quasi-particle picture. More importantly, we demonstrate that the strong electron–vibration coupling is essential to properly describe the overall lineshape of the experimental photoemission spectrum. This cannot be obtained by methods within Born–Oppenheimer approximation. Moreover, we deduce a link between the vibronic states found by our many-body perturbation theory approach and the well-known Jahn–Teller effect.

## Abstract (web summary)

The electron–vibration coupling is essential to describe the photoelectron properties of molecules. Here, the authors show theoretically and experimentally that the electron–vibration coupling is very large in diamonoids, and link the deduced vibronic states to the well-known Jahn–Teller effect.

## Graphical abstract

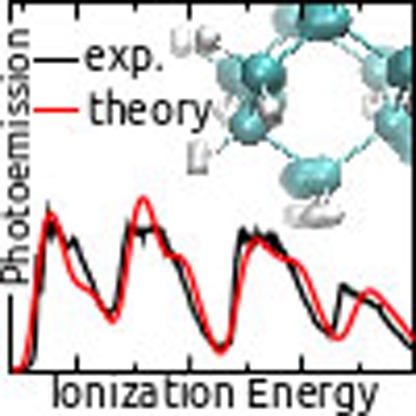

Molecular-sized nanoparticles are applied in diverse areas of basic and applied science, from drug delivery across photovoltaics and quantum information processing, harnessing their unique response to electromagnetic fields^2^,^3^,^4^. Understanding the electronic structure of these systems is an inevitable key step to determine their optical, magnetic and other related properties. One of the most widely used experimental techniques to measure the electronic structure of small nanoparticles and molecules is photoemission spectroscopy. The photoemission spectrum (PES) gives detailed information about the binding energy of the (valence) electrons. Nevertheless, the interpretation of the PES of molecules and nanoparticles may be not as straightforward as for atoms whose spectrum is dominated by sharp peaks^4^. In molecules and nanoparticles, vibrations may play a major role in the ionization process resulting in broad features in PES.

The effect of vibrations in the ionization is often considered within the Born–Oppenheimer approximation where the electrons are decoupled from the vibrations and assumed to be almost perfect quasi-particles (QPs). An approach based on a many-body perturbation theory (MBPT) that goes beyond the Born-Oppenheimer approximation has been recently proposed^5^,^6^,^7^. In this approach both the static and dynamical coupling between electrons and vibrational modes is considered.

Here we show by means of this MBPT approach that in small diamond cages, called diamondoids^9^,^10^,^11^, the electron–vibration coupling leads to the breakdown of the QP picture. As a consequence, the lineshape of their experimental PES can be only reproduced by taking the resultant vibronic states into account. Our MBPT approach is able to predict the Jahn–Teller nature of the corresponding electron states even for deep energy regions. These findings demonstrate the ground breaking impact of our theory in the correct understanding of the most basic photoionization phenomenon in small carbon-based nanoclusters.

## Results

### Photoionization spectrum of diamondoids

**Figure 1:**
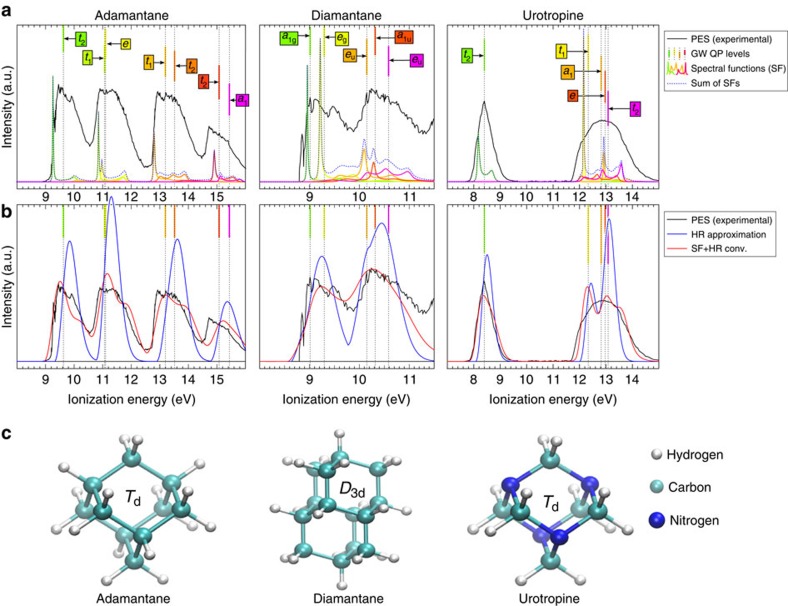
Photoemission spectrum of selected diamondoids: theory and experiment. (**a**) Experimental PES (black curve, adamantane and urotropine from ref. 13, adamantane-urotropine-EXP and diamantane from ref. 14, diamantane-EXP recorded at room temperature) compared with the calculated spectral functions of the electronic QP levels (dotted columns). The different colours represent the different states labelled by their symmetry representation. The corresponding spectral functions (SFs) calculated at zero temperature (see ‘Methods' section) are shown by the same colour code. The sum of the SFs is depicted as a dotted curve. (**b**) Experimental PES (black curve) compared with the broadened QP states (blue curves) and broadened SFs (red curve) where the broadening is calculated by Huang–Rhys (HR) method. The HR spectra are normalized to the area of the corresponding experimental PES and shifted by the zero-point energy difference of the ionized and neutral diamondoids that are 0.21, 0.16 and 0.05 eV for adamantane, diamantane and urotropine, respectively (see ref. 22). (**c**) Geometry of the structures considered with their point group symmetry.

We demonstrate that the interpretation of the PES of selected diamondoids^9^,^12^,^13^, for which the experimental data are available^14^,^15^, in terms of atomic-like, sharp electronic levels is bound to fail. As the geometries of these nano-objects are exactly known, they serve as testbeds to study the accuracy of the standard theoretical tools that aim at describing, from first principles, the electronic structure. Indeed, several density functional theory (DFT)^16^,^17^,^18^, MBPT and Quantum Monte Carlo^19^,^20^,^21^,^22^,^23^ studies have focused on understanding the opto-electronic properties of diamondoids. We consider here the smallest diamondoid with a single diamond cage, called adamantane, characterized by a *T*_d_ symmetry and consisting of only carbon (C) and hydrogen (H) atoms. We also study diamantane, which is composed of two diamond cages with *D*_3d_ symmetry, and a derivative called urotropine with *T*_d_ symmetry, where the C–H units of adamantane are substituted by nitrogen (N) atoms (see Fig. 1c).

The measured and calculated PES of these structures are compared in Fig. 1 at different levels of theory. We start from Kohn–Sham (KS) density functional theory within the local density approximation (LDA). Local (LDA) and other (semi)local DFT functionals provide appropriate zeroth-order approximation for the ground state of the system including the single-particle KS states and energy levels^19^,^18^,^24^. The electron QP levels are calculated within the single-shot GW-method. We note here that fairly convergent QP levels have been obtained for energies down to −15 eV for adamantane and urotropine, and −11 eV for diamantane^23^. Thus, we study the corresponding PES in these photoionization energy ranges.

The effect of vibrations on the PES is treated semiclassically, without dynamical effects, and by including dynamical effects induced by the electron–vibration coupling via MBPT. In the former method, the broadening, or lineshape, of the electron QP levels is described with the Huang–Rhys (HR) method (see ‘Methods' section and Supplementary Methods), which simulates the geometry changes induced by ionization^14^ within the Born–Oppenheimer approximation. We find that the typical total HR factor is *S*≈3 for the considered diamondoids (see Supplementary Fig. 1), which implies that three vibrational modes are involved in the geometry change. In the latter method, instead, the effect of vibrations on the PES is directly included in the spectral function (SF), computed within the recently implemented MBPT approach to the electron–vibration coupling (see refs 5, 6, 7 and the ‘Methods' section for details). The latter method describes polaronic states derived from the potential energy surface of the neutral charge state (equation (11) in the ‘Methods' section) that may be induced on ionization. In our MBPT approach, the resultant SF yields only the lowest energies of these polaronic states with combination of single vibrations belonging to different dynamic distortions of the geometry. The broadening of these polaronic states due to change of the geometry on ionization involving multiple vibration excitations is accounted for by the HR method.

Figure 1 clearly demonstrates that the HR approach of vibration-assisted ionization energies does not account for the experimental PES when the overall lineshape and the broadening of the QPs are considered (see Fig. 1a). Instead, the many-body SFs convolved by HR broadening reproduce remarkably well the overall lineshape of the experimental PES and the broadening of QPs. In particular, the agreement between the experiment and theory is excellent for the adamantane structure in a wide range of photoionization energies: all broad features are correctly described as composed of two local peaks that physically represent coherent packets of electron–vibration states (Fig. 1b). For the other two diamondoids, the dynamical electron–vibration interaction significantly improves the calculated lineshapes compared with the HR method. We emphasize that the broadening is well described not just for the ionization of the highest occupied molecular orbitals but for the deeper states too.

### Analysis of the QP states

In order to understand the calculated PES, we analyse the electronic structure and vibrational modes of the structures we have considered. The lowest unoccupied molecular orbital (LUMO) and the high energy states are very diffuse Rydberg states^18^. As a consequence, their coupling to the vibrational modes is small, and their SFs (see ‘Methods' section) exhibit an almost perfect QP line shape. The calculated QP shifts (see ‘Methods' section) Δ*E*_*n*_ for *n*=LUMO,LUMO+1,…. due to electron–vibration coupling are in the region of 0.05–0.01 eV and the renormalization factors (see ‘Methods' section) *Z*_*n*_ are greater than 0.95. Here, LUMO+1 refers to the index of the state above the LUMO. The situation changes significantly in the case of the highest occupied molecular orbital (HOMO) and lower energy occupied states (HOMO-1, HOMO-2 and so on.), which are the only ones contributing to the PES. These occupied states are localized on the atoms constituting the diamond cages and are strongly coupled to the atomic vibrations. This coupling induces *Z*_*n*_<0.7 for all these states. Values for *Z*_*n*_ far from one clearly point to a potential breakdown of the electron QP picture (in the limit of *Z*_*n*_=0, purely electronic states do not exist). In simple terms, this is confirmed and visualized by the presence of multiple peaks with well-observable intensities appearing in the many-body SFs and in the calculated PES. These states rule out any description in terms of single and well-defined electronic energy states. We note that the breakdown of electron QP states has also been found in other carbon–hydrogen systems, polymers^7^,^8^, and strong renormalization of QP levels led to a decrease of around 7% in the calculated direct gap of bulk diamond^24^.

### SF of adamantane and the Jahn–Teller effect

**Figure 2:**
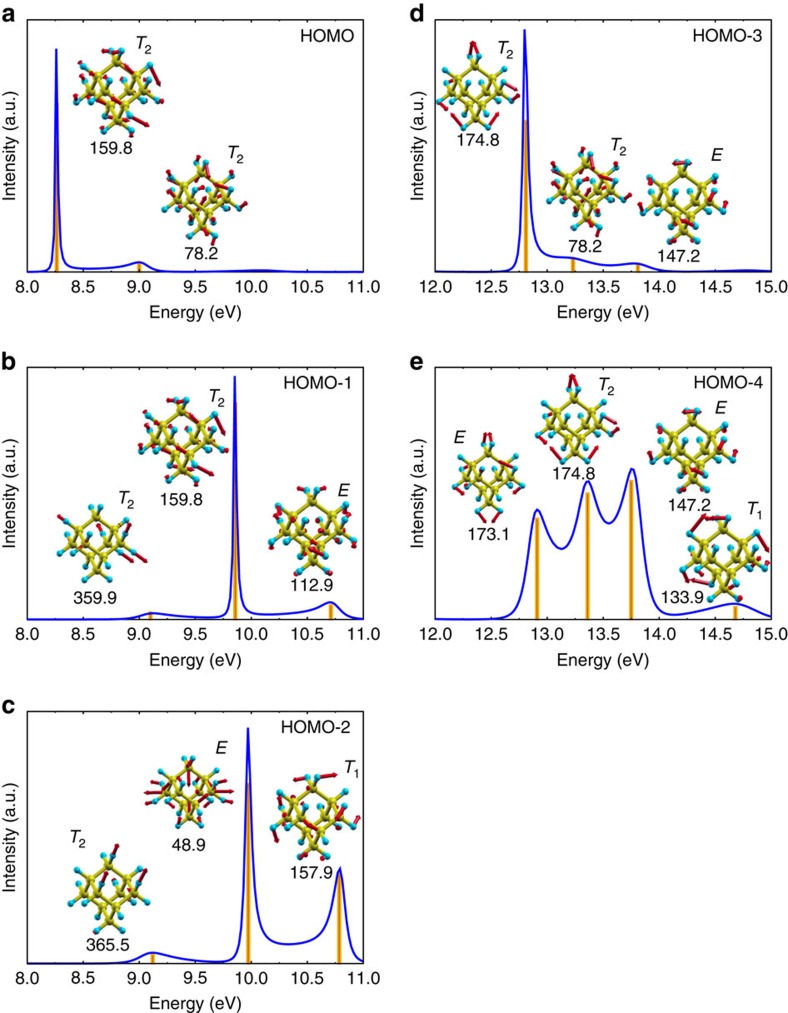
Calculated electron–vibration spectral functions for the labelled occupied states of adamantane. The vibrational modes contributed with the largest extent to the peaks are given in milli-electronvolt unit where the corresponding motion of atoms are depicted by red arrows. We also label the symmetry of vibrational modes. We only show one of the eigenstates of the vibrations in the case of degenerate modes. (**a**) HOMO state with *t*_2_ symmetry. (**b**) HOMO-1 state with *t*_1_ symmetry. (**c**) HOMO-2 state with *e* symmetry. (**d**) HOMO-3 with *t*_1_ symmetry. (**e**) HOMO-4 with *t*_2_ symmetry.

Now we analyse the nature of the calculated SFs represented by the coloured curves in Fig. 1a. We start our analysis with adamantane. The HOMO of adamantane is mostly localized on the C–C *sp*^3^ orbitals forming a triple degenerate *t*_2_ state within *T*_d_ symmetry. Its SF shows two peaks (see Fig. 2a): the main peak is at around 9.25 eV and a second satellite peak at around 10.0 eV. This contributes to around 2 eV broadening of the HOMO, which well reproduces the broadening observed in the experiment (see Fig. 1). The main peak might be associated with the electron QP level that would result in a 0.24 eV shift for the HOMO (for example, the shift between the green dotted line and the maximum peak in the green curve at around 9.5 eV in Fig. 1a). This is a large correction and, more importantly, it corresponds to a QP charge *Z*_*n*_=0.64. This clearly points to the presence of strong dynamical corrections that break the QP picture. Indeed the smaller the QP charge, the larger the participation of the particle in states where the electronic component is minor compared with the vibration excitations. In this case, the state loses a large fraction of its electronic character. Next, we demonstrate the strength of the MBPT approach in the analysis of the complex photoemission spectrum.

**Figure 3:**
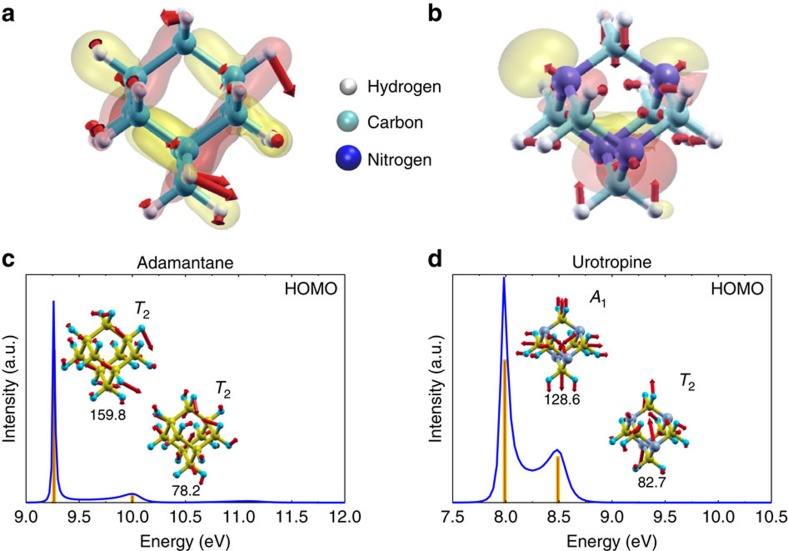
Comparison of the HOMO spectral function for adamantane and urotropine sharing the same *T*_2_ symmetry. The isosurface of the *t*_2(*x*)_ wave functions with an isovalue of ±0.0028 where positive/negative isosurfaces are depicted by red/yellow lobes on the ball-and-stick model of (**a**) adamantane and (**b**) urotropine. The arrows show the displacement of atoms (multiplied by a factor of five for the sake of clarity) going from the the neutral to the ionized state as obtained by KS-DFT LDA calculation (see ref. 22). The corresponding average change of the coordinates of the atoms is larger for adamantane (0.04 Å per atom) than for urotropine (0.02 Å per atom). The vibrational modes contributed dominantly to the given SF are indicated in milli-electronvolt unit and the corresponding motion of the atoms are shown by red arrows in (**c**) adamantane and (**d**) urotropine. We also label the symmetry of vibrational modes. Note the same type of displacements between the dominant vibrational mode in the main peak of SF and the corresponding change in the optimized geometry of the neutral and ionized states. The motion of atoms in these vibration modes for adamantane and urotropine is shown in the Supplementary Movies 1–6 and 7–10, respectively.

The main peak of the HOMO SF comes from the coupling to 36 different vibrational modes in the region of 124–162 meV corresponding mostly to C–C stretch modes. The largest contribution is due to a *T*_2_ vibrational mode at 159.8 meV (see Supplementary Note 1, Supplementary Table 1 and Supplementary Movies 4–6). Most of the other coupled vibration modes are degenerate and an *A*_1_ fully symmetry mode is only weakly coupled. The degenerate vibrational modes coupled to the HOMO of the neutral adamantane move the atoms out of the high symmetry geometry and include the 159.8 meV *T*_2_ mode, which dynamically distorts the high *T*_d_ symmetry geometry to a *C*_3v_ symmetry as demonstrated in Fig. 3a. The DFT calculations of the ionized adamantane in the quasi-static limit result in *C*_3v_ distortion that was partially discussed previously^22^,^25^: the *T*_2_ HOMO state is a Jahn–Teller (JT) system on ionization leaving a hole in the triple degenerate state that may be described as a *t*_2_⊗*T*_2_ vibronic state. We find that the 159.8 meV *T*_2_ vibrational mode that mostly leads to formation of coherent packets of electrons and breaks the QP picture is the same that drives the JT distortion of the ionized adamantane. This finding indicates that the vibronic states found by MBPT induce the JT effect. The link between these effects can be explained by the fact that the linear JT coupling (see Fig. 2 of ref. 26 and references therein) corresponds to the absolute square of the electron–vibration coupling (*g* in Equation 4 in the ‘Methods' section) which is strong for the *t*_2_ HOMO in the neutral adamantane according to our calculations. This strong electron–phonon coupling is manifested in the PES via the calculated SF that contains the amplitude of the transition between the ionized and neutral systems in the MBPT approach (see Lehmann representation of the SF in the ‘Methods' section). This shows that our MBPT approach indicates the JT nature of the ionized systems. This is a particularly important finding as this can be extended to the degenerate states lying below the HOMO level.

By following this argument other important conclusions can be drawn in the interpretation of the calculated SF and the experimental PES. The second peak of the HOMO SF corresponds to a satellite that originates from the coupling to 43 different degenerate vibrational modes with energy of 78–158 meV. These states correspond to the twist and bending modes of C atoms. The largest contribution belongs to the 78.2-meV *T*_2_ twist mode (see Supplementary Note 1, Supplementary Table 1 and Supplementary Movies 1–3). These twist modes do not appear in bulk diamond but only in the quasi zero-dimensional diamond cages and lead to a dynamical symmetry distortion that is peculiar for these systems. Analogue to the main HOMO SF peak, this satellite HOMO SF peak can be associated with the motion of atoms in the ionized system that distorts the *T*_d_ symmetry due to JT effect. We refer to it as anomalous JT distortion by following Patrick and Giustino^25^. Remarkably, our MBPT approach is able to identify the origin of the satellite HOMO peak in PES and implies a link to the JT effect. We further note that these twist modes contribute to the second local maximum in the first broad feature of the PES of adamantane at around 10 eV (for example, Figs 1a and 2a).

### Comparison of adamantane and urotropine

At this point, it is intriguing to compare the *t*_2_ HOMO states of adamantane and urotropine that share the same *T*_d_ symmetry but show distinct shape of PES for HOMO (see Fig. 1b). We compare the HOMO SFs for adamantane and urotropine in Fig. 3. The HOMO in urotropine is strongly localized on the four N atoms. It couples to 39 (20) different vibrational modes in the energy region of 56 (56) meV to 93 (129) meV for the main (satellite) peak (see Supplementary Note 1 and Supplementary Table 3). This results in about 1.5 eV broadening of HOMO in urotropine that is about 0.5 eV smaller than that in adamantane. Furthermore, the main and the second peaks originate from a coupling with a fully symmetric *A*_1_ vibrational mode of 128.6 meV (see Supplementary Movie 10). In the second peak, the contribution of a *T*_2_ vibrational mode of 82.7 meV may imply a JT distortion (see Supplementary Movies 7–9). In the main peak, the coupling to many degenerate vibrational modes appears but only with a minor weight. We find by quasi-static DFT calculations on the ionized urotropine that the JT distortion is minor and the atoms move with a smaller amplitude upon ionization than those in adamantane. In other words, the JT effect is smaller in urotropine than in adamantane. The proposed link between MBPT SF of the neutral system and the JT effect of the ionized system is in line with this finding: a stronger JT effect results in a wider electron–vibration-related broadening in PES. This feature is well captured by our MBPT approach.

### Analysis of deep QP states of adamantane

We continue the analysis of PES for adamantane in Fig. 1. As we mentioned earlier, the second local maximum at around 10 eV can be explained by the satellite peak of the HOMO. However, it is evident from the dotted curve in Fig. 1a that low-energy satellite peaks of the closely lying HOMO-1 and HOMO-2 states also contribute to that. The HOMO-1 *t*_1_ and HOMO-2 *e* states are degenerate and also subject to the JT distortion upon ionization. Indeed, the low-energy satellite peak, the main peak and the high-energy satellite peak originate mostly from the coupling to different degenerate vibrational modes (see Fig. 2b,c, respectively) that dynamically distort the *T*_d_ symmetry in both cases. The main peak and the high-energy satellite peak of HOMO-1 and HOMO-2 states contribute to the shape of the second broad feature in the PES of adamantane. Finally, similar conclusion can be drawn for the third and fourth broad features in PES. The third broad feature corresponds to the HOMO-3 *t*_1_ and HOMO-4 *t*_2_ states that are triple degenerate and subject to JT distortion again. Their SFs are shown in Fig. 2d,e, respectively. As the broadening of these states due to electron–vibration coupling is very large (above 2.5 eV), their SFs strongly overlap despite the relatively large QP energy gap of 0.4 eV (for example, orange dotted lines and the corresponding orange curves under the third broad feature of black curve in Fig. 1a). Particularly, the four peaks that are visible in the SF of HOMO-4 clearly demonstrate the breakdown of the electron QP picture. We found that more than 100 different vibrational modes within an energy range of 37–365 meV couple to this electronic state and contribute to the corresponding peaks in the SF. We note that the dynamic distortion of the 147.2 meV mode in the HOMO-3 SF couples to the HOMO-4 state whereas the dynamic distortion of the 173.1 meV mode in the HOMO-4 SF couples to the HOMO-3 state. The final lineshape of the calculated PES is very asymmetric and it is well represented by the calculated SFs convolved by HR method. A very similar process is responsible for the asymmetry of the fourth broad feature originating from the HOMO-5 and HOMO-6 states and the broad features in urotropine composed from HOMO-1 to HOMO-4 states.

### Signature of pseudo Jahn–Teller effect in diamantane

Next, we analyse the PES of diamantane with *D*_3d_ symmetry. The calculated SFs of diamantane HOMO and the lower energy states are very broad and create an almost featureless PES, in good agreement with the experiment (Fig. 1b). The HOMO and the HOMO-1 states, the fully symmetric non-degenerate *a*_1g_ and the double degenerate *e*_g_ states are localized mainly on the C atoms. The HOMO-2, HOMO-3 and HOMO-4 states are characterized by *e*_u_, *a*_1u_ and *e*_u_ symmetry, respectively.

First, we focus our attention on the HOMO and HOMO-1 and their interaction. Although *a*_1g_ and *e*_g_ states are principally distinct in nature, their SFs look very similar (see the green and yellow curves with a maximum at around 9.0 eV in Fig. 1a). By recalling our proposal for a link between the strength of JT coupling and the width of broadening in PES based on the analysis of the HOMO in adamantane and urotropine, this might be surprising since the non-degenerate *a*_1g_ state is stable against JT distortion whereas the double degenerate *e*_g_ state is not.

**Figure 4:**
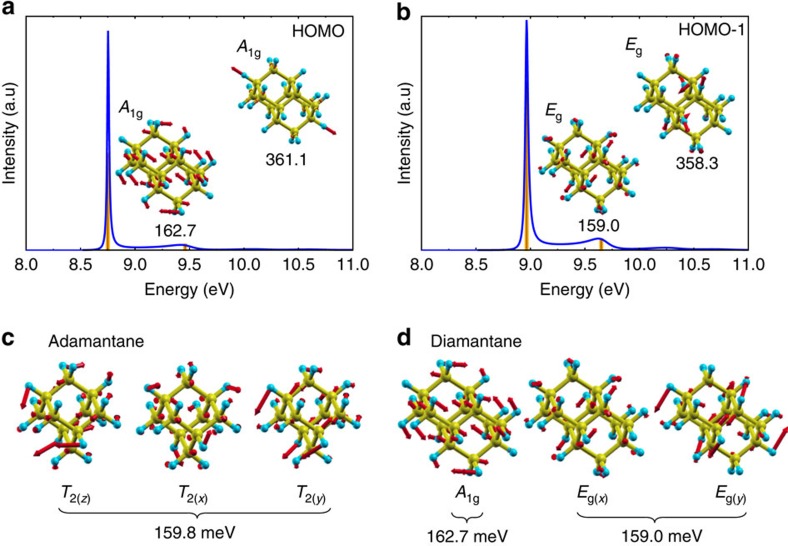
Comparison of the HOMO and HOMO-1 spectral functions for diamantane possessing *a*_1g_ and *e*_g_ symmetry, respectively. The vibration modes contributed dominantly to the largest peak of SF are indicated in milli-electronvolt unit with the corresponding symmetry labels where the motion of the atoms are shown by red arrows for (**a**) HOMO and (**b**) HOMO-1 levels. Comparison of the vibrational modes in (**c**) adamantane and (**d**) diamantane that couples to the highest occupied molecular orbitals. The motion of atoms in these vibration modes for adamantane and diamantane is shown in the Supplementary Movies 4–6 and 11–13, respectively.

We anticipate that this phenomenon is a manifestation of the so-called pseudo-Jahn–Teller effect^27^. We explain this feature in conjunction with the HOMO of adamantane and their relation to the valence band edge of bulk diamond. The valence band edge of diamond at the Γ-point is a triple-degenerate *t*_2g_ under *O*_h_ space group neglecting the spin–orbit interaction. This *t*_2g_ state is built up from the linear combination of *sp*^3^ hybrid valence orbitals of carbon atoms. Adamantane, as a single cage of diamond, forms very similar triple-degenerate HOMO orbital with a correspondence of *O*_h_:*t*_2g_→*T*_d_:*t*_2_. Diamantane with two diamond cages forms a lower symmetry, so *O*_h_:*t*_2g_→*D*_3d_:*a*_1g_+*e*_g_; the original triple-degenerate state of diamond ‘HOMO' orbital splits to a non-degenerate and a double degenerate state. Nevertheless, this splitting does not originate from the chemical nature of the bonds but from the low symmetry of ions, thus these states have hidden connection, and they may be considered as pseudo-degenerate states. This is the reason why the SFs of adamantane's HOMO *t*_2_ and diamantane's HOMO *a*_1g_ and HOMO-1 *e*_g_ orbitals have very similar SF line-shapes. The 159.8 meV *T*_2_ mode, the most responsible for the JT distortion of adamantane HOMO (main peak in SF), appears as 162.7 meV *A*_1g_ mode for HOMO (see Supplementary Movie 13) and 159.0 meV *E*_g_ mode for HOMO-1 in diamantane (see Supplementary Movies 11,12) where similar correspondence can be derived between these vibrational modes (see Fig. 4) as for their electronic states. Particularly, the *z*-component of the triple-degenerate *T*_2_ vibrational mode of adamantane is mimicked by the fully symmetric *A*_1g_ vibration mode in diamantane. These *A*_1g_ and *E*_g_ vibrational modes distort the diamond cages of diamantane similarly to the corresponding components of the *T*_2_ vibrational mode of adamantane (Fig. 4b). Thus, *A*_1g_ vibration-related motion of atoms represents a pseudo-JT distortion of the diamond cages in diamantane upon ionization. To summarize, the vibrational modes that lead to formation of coherent packets of electrons and vibrational modes and break the QP picture are the same that drive the pseudo-JT distortion. The hidden connection between the electronic states can be analysed by the corresponding MBPT SFs.

We discuss further the PES of diamantane. The second peak in the SF of HOMO and HOMO-1 states of diamantane shows a similar anomalous JT distortion to that of the HOMO of adamantane. Going further with the group theory analysis, the HOMO-2 and HOMO-3 *e*_u_ and *a*_1u_ states have a correspondence to the adamantane's HOMO-1 *t*_1_ state, while the HOMO-4 *e*_u_ state has a relation to the adamantane's HOMO-2 *e* state. However, as the symmetry of the two *e*_u_ states are the same in diamantane, these electronic states can mix the character of the ascendant *t*_1_ and *e* states of adamantane. This also results in a much larger number of vibrational modes that couple these two *e*_u_ electronic states compared with the coupling of the *t*_1_ and *e* states in adamantane (for example, Supplementary Table 1 versus Supplementary Table 2). The computed PES shows an almost featureless shape at around 10.3 eV for diamantane, in contrast to the corresponding spectrum at around 11.0 eV for adamantane (see Fig. 1b). Thus, the difference in the broadening and the lineshape of the two diamondoids could be well explained by the analysis of their orbitals, coupling of states via vibrations, and the proposed JT effects upon ionization.

## Discussion

We note that the fine structure of the experimental PES consisting of sharp features requires further discussion. Our HR spectrum on the diamondoids suggests (see red curves in Supplementary Fig. 1) that the fine structure could be related to the ionization-induced changes in the geometry. However, the accurate calculation on the strength of this contribution requires to consider the dynamical and geometry-induced effects on the same footing. This is equivalent to extend the present MBPT approach to include multiple vibrational excitations in the form of a vertex correction. Our MBPT approach has to be extended to capture multiple vibrational excitations, and we anticipate that vertex corrections can account for this. Inspired by the success of the present combined HR+MBPT approaches, we are actively pursuing the development of this more comprehensive theory.

In conclusion, we demonstrated on small nanodiamonds that the electron–vibration coupling results in the breakdown of the electron QP picture for their occupied states even at zero temperature. This has serious consequences on the features of their photoemission spectra, which cannot be predicted by semiclassical vibration broadening of electron states. We found indications in our MBPT approach for the fingerprint of different JT distortions. Our results imply that understanding of all the experimental features in the photoexcitation or photoionization of small nanoparticles needs a detailed investigation of electron–vibration coupling. We showed that our fully *ab initio* MBPT approach is capable of studying realistic systems and analyse the role of vibrations in their photoionization processes. This makes possible to design structures where the vibrations are manipulated to achieve the desired opto-electronic properties, which is of immediate importance in the field of superconductors, photovoltaic materials, colour centres in nanodiamonds^4^ and related materials^28^ for nanometrology.

## Methods

### Ground-state calculations

The ground-state geometry and electronic structure of the diamondoids were computed by plane wave supercell KS-DFT calculations as implemented in the Quantum Espresso (QE) software package^29^. We used a simple cubic simulation box and a diamondoid–diamondoid separation of about 1 nm to avoid the spurious interaction between the periodic images. For diamantane, we applied a hexagonal box to have a simulation box commensurable with its point group symmetry. The core electrons were taken into account by using norm-conserving Troullier–Martins pseudopotentials^30^. We took the standard Troullier–Martins pseudopotentials for each atom from the QE database except for nitrogen where we applied a relatively soft pseudopotential. We found that a kinetic energy cutoff of 45 Ry for plane wave expansion was sufficient to converge the band gap of the selected diamondoids. We applied *ab initio* LDA within KS-DFT to calculate the total energy and the KS spectrum^31^,^32^. The geometry of the diamondoids was optimized by minimizing the Hellmann–Feynmann forces below the threshold of 0.001 Ry per Bohr. In the geometry optimization procedure, we applied 80 Ry kinetic energy cutoff. We aligned single particle energies to the vacuum level by using the Martyna–Tuckerman method^33^ as implemented in QE. The vibrational frequencies were computed by density functional perturbation theory^34^. The calculations were performed with a strong threshold of 10^−16^ Ry to achieve the self-consistent total energy in order to obtain accurate vibrational modes.

### The implemented HR method to calculate vibration broadening

The rotations of single diamondoids and their collisions with other cages cause a broadening of about 20 meV (ref. 14) that does not explain the two orders magnitude larger broadening in the experimental PES. This is due to the relatively strong electron–vibration coupling where high energy vibration states with *n*>0 quantum number contribute to the PES. The vibration-assisted ionization spectrum can be calculated by the HR method^35^ within the Franck–Condon approximation that is explained briefly as follows. The key equation is the SF of electron–phonon coupling^36^


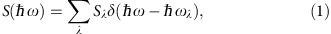


where the sum is over all vibration modes *λ* with frequencies *ω*_*λ*_, and *S*_*λ*_ is the partial HR factor for the mode *λ*. *S*_*λ*_ basically is the strength of the electron–vibration coupling of vibration *λ*. This can be defined as


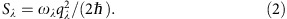


where


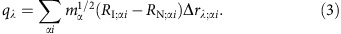


*m*_α_ is the mass of atom *α*, *i*=*x*,*y*,*z*, *R*_{I,N};*αi*_ is the equilibrium position in the ionized (I) and neutral (N) state and Δ*r*_*λ;αi*_ is normalized vector that describes the displacement of the atom *α* along the direction *i* in the vibration mode *λ*. On ionization of the diamondoids, the optimized geometry changes from neutral to the ionized state within our Born–Oppenheimer approximation calculated by the chosen DFT. The vibration modes of the neutral diamondoids are calculated by the density functional perturbation theory method. The total HR factor *S* is obtained by 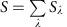. Generally, if *S*<<1 then the spectrum is very sharp and mostly localized at the position of the QP energy. If *S*>10, then the spectrum is very broad, featureless and can be well described with a symmetrical Gaussian function. We found that the typical value for diamondoids, *S*∼3, leads to a more complicated structure. The spectrum consists of a few sharp features lying on a broad background and the shape of the spectrum is non-symmetric. The *S*∼3 implies that three vibration quanta should contribute to the spectrum. To create a simplified curve for broadening, we replaced the structured HR SF with a continuous envelope function. In the Supplementary Methods and the corresponding Supplementary Fig. 1, we give a more detailed description about this methodology and the results on the selected diamondoids.

### The dynamical approach to the electron–vibration problem

Our methodology has been derived in detail in refs 6, 7. Here, we only briefly summarize the basic assumptions and the key equations. Since we have finite objects within the supercell formalism, we use the *k*=0 in the Brillouin zone and thus the momentum transfer *q* is also set to zero.

Using finite temperature MBPT, one can derive the self energy of an electron due to its interaction with vibrational modes. We consider the lowest-order self-energy terms: Fan-term (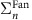) and DW-term (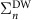) (refs 6, 7). Both depend on the temperature (*T*) through the boson occupation number of the vibration states (*B*(*ω*_*λ*_)):


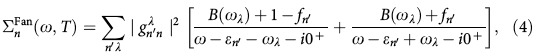



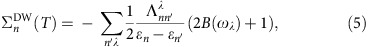


Here, *n* and 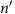 are the KS electron states with KS energies *ɛ*_*n*_ and 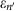, respectively. *f*_*n*_ is the Fermi function of electrons. Since the diamondoids have large gaps, *f*_*n*_ is 1(0) for occupied (unoccupied) states, respectively. 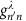 is the electron–vibration matrix element between states *n* and 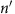 due to the vibrational mode *λ* and energy *ω*_*λ*_. This is a linear combination of 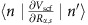 weighted by the amount they contribute to each vibrational mode, where 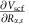 is the change in the self-consistent potential due to the displacement *R*_*α*,*S*_ of atom *s* along the cartesian direction α. We compute the first-order derivatives of the KS potential using density functional perturbation theory^34^. The second-order derivatives appearing in equation (5) (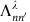) can be turned into products of first-order derivatives by using translational invariance. In the summations over 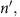 we used 200 states and checked convergence by using 300 states for selected diamondoids. In the summations over *λ*, we did not include the first six modes corresponding to translations and rotations of the diamondoids.

The temperature-dependent Green-function due to electron–vibration interaction can be written as


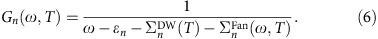


By Taylor expanding the Fan term around the unperturbed energies, we can also define perturbative corrections to the electron QP states:


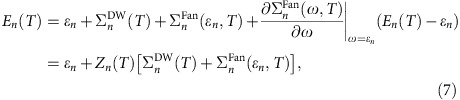


where the renormalization factor *Z*_*n*_(*T*) reads


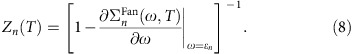


The QP shift Δ*E*_*n*_(*T*) due to electron–vibration interaction may be simply defined as Δ*E*_*n*_(*T*)=*E*_*n*_(*T*)−*ɛ*_*n*_. We note that the PES for diamondoids was measured at room temperature. For adamantane, we checked that bosonic occupation of vibration states indeed do not change the results going from zero kelvin up to room temperature. Since N–C, C–C and C–H vibrational modes have much higher energies than the room temperature energy of 26 meV, only the ground-state vibration states have non-zero occupations, and thus the calculated properties do not show measurable temperature dependence in the region of zero kelvin and room temperature. Therefore, we omit the temperature dependence in the QP shift Δ*E*_*n*_≡Δ*E*_*n*_(*T*) and the renormalization factor *Z*_*n*_≡*Z*_*n*_(*T*).

We can also compute the SF, which is defined as


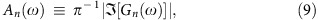


where *G*_*n*_(*ω*) is taken from equation (6) by omitting the temperature. These SFs are plotted for a given *n* KS state in Fig. 2 for adamantane from which the PES was derived as shown in Fig. 1b. We note here that equation (9) does not include transition matrix element effects and thus our method of computing PES corresponds to a constant, energy-independent transition matrix element approximation.

For the analysis of the contribution of the vibrations to the corresponding SFs for a given KS state, we use the Lehmann representation,


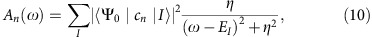


where *E*_*I*_ is the energy of the true eigenstate (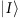) of the system and *η* is a small infinitesimal number. 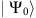 is the exact ground state and *c*_*n*_ is the electron annihilation operator. Here, we again omit for simplicity the temperature dependence. By definition, *E*_*I*_ are the real poles of *G*_*n*_(*ω*).

In ref. 7, it was shown that the states 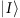 are mixed packets of electrons and vibrations. Moreover, their internal structure was found by showing that the electron–vibration problem can be fully rewritten as the solution of an effective Hamiltonian. The solution of this problem allows to introduce an explicit expression for the interacting states 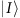:


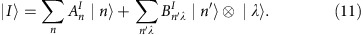



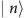
 is the purely electronic state obtained by creating an electron in the *n-*th KS level. 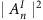 is the corresponding probability to find the electron–vibration state along 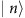. 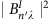, instead, is the probability to find the fully interacting state in a mixed electronic 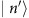 and vibrational *λ* state (see refs 7, 37). These coefficients can be found by diagonalizing the electron–vibration Hamiltonian on the basis of equation (11). This tool was applied to analyse the contribution of vibrations *λ* to the given peaks in the SFs, for instance, as depicted in Fig. 2 for adamantane.

From the Lehmann representation, it follows that the Green-function represents the dynamical evolution of the charged state 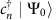. This situation is quite similar to the physics described in the Jahn–Teller effect. The difference is that, in the present case, the atoms are allowed to move only within the harmonic approximation. Nevertheless, as done in ref. 26, the analysis of the 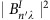 terms can be used to deduce the most intense phonon modes (and consequently, atomic displacement directions) that contribute to the formation of the coupled electron–vibration states.

The complex structure of the 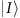 state, then, induces the wealth of structures that are crucial to explain the PES and, at the same time, provides a sound and clear indication of the most intense modes that contribute to the physics of the Jahn–Teller effect.

## Additional information

**How to cite this article:** Gali, A. *et al.* Electron–vibration coupling induced renormalization in the photoemission spectrum of diamondoids. *Nat. Commun.* 7:11327 doi: 10.1038/ncomms11327 (2016).

## Supplementary Material

Supplementary Information Supplementary Figure 1, Supplementary Tables 1-3, Supplementary Note 1 and Supplementary Methods

Supplementary Movie 1 The video shows the vibration mode T_2(x)_ at 78.2 meV for adamantane. The carbon and hydrogen atoms are depicted by brown and white balls, respectively.

Supplementary Movie 2 The video shows the vibration mode T_2(y)_ at 78.2 meV for adamantane. The carbon and hydrogen atoms are depicted by brown and white balls, respectively.

Supplementary Movie 3 The video shows the vibration mode T_2(z)_ at 78.2 meV for adamantane. The carbon and hydrogen atoms are depicted by brown and white balls, respectively.

Supplementary Movie 4 The video shows the vibration mode T_2(x)_ at 159.8 meV for adamantane. The carbon and hydrogen atoms are depicted by brown and white balls, respectively.

Supplementary Movie 5 The video shows the vibration mode T_2(y)_ at 159.8 meV for adamantane. The carbon and hydrogen atoms are depicted by brown and white balls, respectively.

Supplementary Movie 6 The video shows the vibration mode T_2(z)_ at 159.8 meV for adamantane. The carbon and hydrogen atoms are depicted by brown and white balls, respectively.

Supplementary Movie 7 The video shows the vibration mode T_2(x)_ at 82.7 meV for urotropine. The carbon, nitrogen and hydrogen atoms are depicted by brown, blue and white balls, respectively.

Supplementary Movie 8 The video shows the vibration mode T_2(y)_ at 82.7 meV for urotropine. The carbon, nitrogen and hydrogen atoms are depicted by brown, blue and white balls, respectively.

Supplementary Movie 9 The video shows the vibration mode T_2(z)_ at 82.7 meV for urotropine. The carbon, nitrogen and hydrogen atoms are depicted by brown, blue and white balls, respectively.

Supplementary Movie 10 The video shows the vibration mode A_1_ at 128.6 meV for urotropine. The carbon, nitrogen and hydrogen atoms are depicted by brown, blue and white balls, respectively.

Supplementary Movie 11 The video shows the vibration mode E_g(x)_ at 159.0 meV for diamantane. The carbon and hydrogen atoms are depicted by brown and white balls, respectively.

Supplementary Movie 12 The video shows the vibration mode E_g(y)_ at 159.0 meV for diamantane. The carbon and hydrogen atoms are depicted by brown and white balls, respectively.

Supplementary Movie 13 The video shows the vibration mode A_1g_ at 162.7 meV for diamantane. The carbon and hydrogen atoms are depicted by brown and white balls, respectively.
