# Supplementary material for: Electron–vibration coupling induced renormalization in the photoemission spectrum of diamondoids
Source: Nat Commun. 2016 Apr 22;7:11327. doi: 10.1038/ncomms11327 (PMC4844694; doi:10.1038/ncomms11327)
Supplement: Supplementary Information — Supplementary Figure 1, Supplementary Tables 1-3, Supplementary Note 1 and Supplementary Methods [file ncomms11327-s1.pdf]

## Supplementary Figures

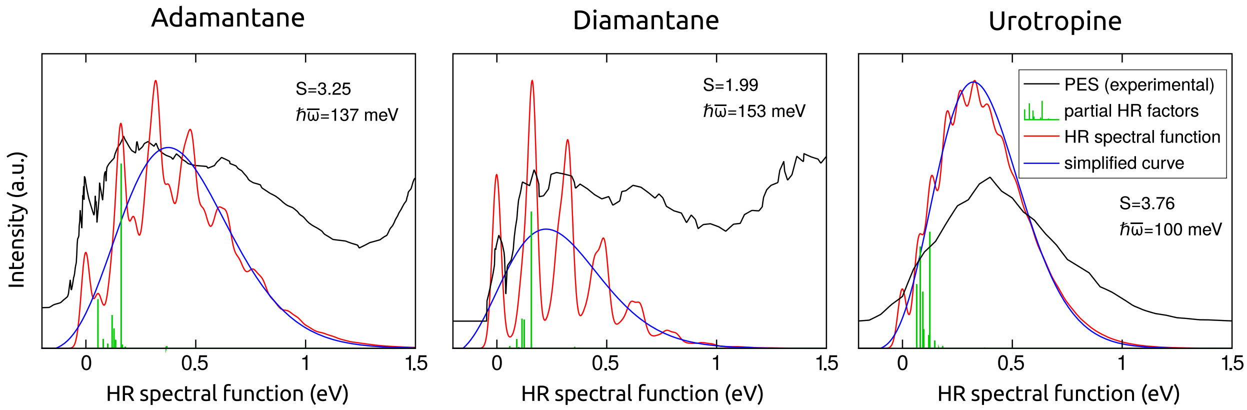


Supplementary Figure 1. **Broadening of the quasi-particle levels.** The calculated broadening of the HOMO levels by using the Huang-Rhys (HR) approximation (red curve) as obtained from the calculated partial HR factors (green peaks). The calculated total HR factors $S$ are shown for each diamondoid. An extra Gaussian broadening of 20 meV was also applied that simulates the rotation and collision effects. The sharp peaks occur due to the average 137-meV, 153-meV and 100-meV vibration modes in adamantane, diamantane, and urotropine, respectively, that are coupled stronger to the HOMO than other modes. We also plot the experimental photoemission spectrum around HOMO of the corresponding diamondoids for comparison (black curve) that is aligned arbitrarily to the calculated HR spectral function. The pure quasi-particle peak and the first two or three sharp peaks associated with the strongly coupled vibration mode is visible for adamantane and diamantane. The experimental spectrum for urotropine is not well structured. A simplified function (blue curve) describes the broadening of the calculated spectrum without showing this fine structure of the photoemission spectra. See Supplementary Methods for more details.

## Supplementary Tables

Supplementary Table 1. Adamantane related vibration modes and their coupling to the given electronic state.

| | | | | | |
|---|---|---|---|---|---|
| HOMO (eV) | SF 26-28, t2 | | | | |
| № of peak | coupled state | mode | symmetry | frequency (meV) | residual |
| 1 (9.00-9.50) | 26-28 | 46-48 | T2 | 159.8 | 0.07 |
| 2 (9.75-10.25) | 21-22 | 15-17; 27-29; 36; 44 | T2; T2; T1; T1 | 78.2; 118.8; 133.9; 157.9 | 0.150; 0.110; 0.070; 0.064 |
| HOMO-1 (eV) | SF 23-25, t1 | | | | |
| № of peak | coupled state | mode | symmetry | frequency (meV) | residual |
| 1 (9.75-10.25) | 23-25 | 41; 63-65 | E; T2 | 147.2; 359.9 | 0.040; 0.090 |
| 2 (10.75-11.25) | 23-25; 21-22 | 41; 46-48 | E; T2 | 147.2; 159.8 | 0.036; 0.039 |
| 3 (11.50-12.00) | 15-17 | 25-26; 31-33 | E; T1 | 112.9; 128.8 | 0.200; 0.100 |
| HOMO-2 (eV) | SF 21-22, e | | | | |
| № of peak | coupled state | mode | symmetry | frequency (meV) | residual |
| 1 (9.75-10.25) | 23-25 | 76-78 | T2 | 365.5 | 0.180 |
| 2 (10.75-11.25) | 21-22 | 10-11; 30; 62 | E, A1; A1 | 48.9; 128.1; 177.3 | 0.036; 0.020; 0.020 |
| 3 (11.50-12.00) | 15-17 | 44; 46-48; 52-54 | T1; T2; T2 | 157.9; 159.8; 164.2 | 0.160; 0.090; 0.100 |
| HOMO-3 (eV) | SF 18-20, t1 | | | | |
| № of peak | coupled state | mode | symmetry | frequency (meV) | residual |
| 1 (12.75-13.00) | 23-25; 21-22 | 57; 59-61; 74 | E; T2; T1 | 173.1; 174.8; 365.2 | 0.080; 0.090; 0.200 |
| 2 (13.00-13.50) | 18-20; | 15-17; 67-68 | T2; E | 78.2; 360.5 | 0.045; 0.016 |
| 3 (13.50-14.00) | 15-17 | 7-9; 11; 22-24; 42 | T1; E; T1; E | 37.2; 48.9; 105.9; 147.2 | 0.050; 0.140; 0.027; 0.051 |
| HOMO-4 (eV) | SF 15-17, t2 | | | | |
| № of peak | coupled state | mode | symmetry | frequency (meV) | residual |
| 1 (∼13.00) | 18-20 | 46-48; 57-58; 59-61 | T2; E; T2 | 159.8; 173.1; 174.7 | 0.039; 0.040; 0.034 |
| 2 (∼13.50) | 15-17 | 25-26; 41-42; 60 | E; E; T2 | 112.9; 147.2; 174.8 | 0.030; 0.050; 0.060 |
| 3 (∼14.00) | 15-17 | 12-14; 18; 25-26; 41-42 | T2; A1; E; E | 52.8; 94.9; 112.9; 147.2 | 0.025; 0.027; 0.026; 0.043 |
| 4 (∼14.50) | 11-13 | 19-21; 34-36; 40 | T2; T1; A2 | 99.9; 133.9; 134.2 | 0.057; 0.070; 0.060 |

Supplementary Table 2. Diamantane related vibration modes and their coupling to the given electronic state.

| | | | | | |
|---|---|---|---|---|---|
| HOMO (eV) | SF 38, a1g | | | | |
| № of peak | coupled state | mode | symmetry | frequency (meV) | residual |
| 1 (∼8.5-9.5) | 38 | 68, 94 | A1g, A1g | 162.65, 361.13 | 0.18, 0.20 |
| 2 (∼9.5-9.8) | 36-37; 38 | 15-16, 53-54; 68 | Eg, Eg; A1g | 50.9, 142.67; 162.65 | 0.05, 0.05; 0.09 |
| HOMO-1 (eV) | SF 36-37, eg | | | | |
| № of peak | coupled state | mode | symmetry | frequency (meV) | residual |
| 1 (∼8.8-9.8) | 36-37; 38 | 61-62; 84-85 | Eg; Eg | 159.0; 358.3 | 0.06; 0.07 |
| 2 (∼9.5-10.2) | 36-37 | 61-62 | Eg; Eg | 159.0 | 0.06 |
| HOMO-2 (eV) | SF 34-35, eu | | | | |
| № of peak | coupled state | mode | symmetry | frequency (meV) | residual |
| 1 (∼9.9-10.4) | 34-35 | 99-100 | Eg | 365.2 | 0.10 |
| 2 (∼10.4-11.1) | 33 | 32-33 | Eg | 114.86 | 0.03 |
| 3 (∼10.8-11.8) | 31-32 | 26, 29, 36 | A1g, A2g, A1g | 99.7, 107.3, 120.5 | 0.16, 0.38, 0.11 |
| HOMO-3 (eV) | SF 33, a1u | | | | |
| № of peak | coupled state | mode | symmetry | frequency (meV) | residual |
| 1 (∼9.9-10.4) | 33 | 88 | A1g | 358.97 | 0.140 |
| 2 (∼10.4-10.9) | 31-32; 33 | 99-100; 81 | Eg; A1g | 365.2; 175.6 | 0.06; 0.04 |
| 3 (∼10.9-11.4) | 31-32 | 61-62, 53-54 | Eg, Eg | 159, 142.7 | 0.05, 0.04 |
| HOMO-4 (eV) | SF 31-32, eu | | | | |
| № of peak | coupled state | mode | symmetry | frequency (meV) | residual |
| 1 (∼10.3-10.8) | 31-32 | 68 | A1g | 162.6 | 0.18 |
| 2 (∼10.7-11.2) | 30 | 99-100 | Eg | 365.2 | 0.61 |
| 3 (∼11.1-11.6) | 30 | 90-91, 99-100 | Eg, Eg | 360.2, 365.2 | 0.12, 0.25 |

Supplementary Table 3. Urotropine related vibration modes and their coupling to the given electronic state.

| HOMO (eV) | SF 26-28, t2 | | | | |
|---|---|---|---|---|---|
| № of peak | coupled state | mode | symmetry | frequency (meV) | residual |
| 1 (∼7.8-8.2) | 26-28 | 31 | A1 | 128.6 | 0.210 |
| 2 (∼8.3-8.8) | 26-28 | 15, 31 | T2, A1 | 82.7, 128.6 | 0.041, 0.100 |
| HOMO-1 (eV) | SF 22-24, t1 | | | | |
| № of peak | coupled state | mode | symmetry | frequency (meV) | residual |
| 1 (∼12.0-12.4) | 22-24 | 57-59, 60, 61-63 | T2, A1, T1 | 359.0, 360.3, 365.2 | 0.080, 0.070, 0.090 |
| 2 (∼12.5-12.9) | 20-21; 22-24 | 64-66; 54 | T2; A1 | 365.3; 175.5 | 0.020; 0.040 |
| 3 (∼13.3-13.7) | 17-19 | 7-9, 25-27 | T1, T2 | 46.0, 125.9 | 0.060, 0.040 |
| HOMO-2 (eV) | SF 20-21, e | | | | |
| № of peak | coupled state | mode | symmetry | frequency (meV) | residual |
| 1 (∼12.0-12.4) | 25 | 19-21, 25-27 | T2, T2 | 102.1, 125.9 | 0.110, 0.370 |
| 2 (∼12.6-13.0) | 20-21 | 61-63 | T1 | 365.2 | 0.18 |
| 3 (∼13.2-13.6) | 17-19 | 35-37, 44-45 | T2, E | 151.5, 162.3 | 0.018, 0.020 |
| HOMO-3 (eV) | SF 25, a1 | | | | |
| № of peak | coupled state | mode | symmetry | frequency (meV) | residual |
| 1 (∼12.8-13.2) | 25; 22-24 | 31; 60, 61-63 | A1; A1, T1 | 128.6; 360.3, 365.2 | 0.070; 0.130, 0.130 |
| 2 (∼13.3-13.7) | 22-24 | 7-9; | T1 | 46.0 | 0.130 |
| 3 (∼14.1-14.6) | 17-19 | 15-17, 19-21 | T2, T2 | 82.7, 102.1 | 0.090, 0.130 |
| HOMO-4 (eV) | SF 17-19, t2 | | | | |
| № of peak | coupled state | mode | symmetry | frequency (meV) | residual |
| 1 (∼12.0-12.4) | 22-24; 25 | 35-37, 46-48; 33 | T2, T2; E | 151.5, 163.5; 128.9 | 0.150, 0.060; 0.210 |
| 2 (∼12.6-13.0) | 20-21 | 55-56, 60 | E, A1 | 358.8, 360.3 | 0.120, 0.110 |
| 3 (∼13.2-13.6) | 17-19 | 15-17, 30 | T2, T1 | 82.7, 126.3 | 0.022, 0.030 |

## Supplementary notes

### Supplementary note 1

### Analysis of the spectral function and the corresponding vibration modes

Below we give the most important data for the analysis of the spectral functions for adamantane (Supplementary Table 1), diamantane (Supplementary Table 2), and urotropine (Supplementary Table 3), respectively. The tables can be interpreted as follows. The first column labels the electronic level such as the highest occupied molecular orbital (HOMO), the level below it (HOMO−1), etc. In the spectral function the breakdown of the electron quasi-particle (QP) picture may result in several peaks for a given electronic level. For instance, there are two peaks for HOMO in adamantane (see Supplementary Table 1). The peaks are labeled by № symbol of 1, 2, etc., and the energetic position of these broadened peaks (in eV) are also shown beside the index of the peak in parenthesis. In the top row of the second column we provide the index of the given electronic state ('SF') together with its symmetry. In the case of degenerate state, all the degenerate states'

indexes are given (e.g., SF 26-28 in adamantane means that the HOMO is triple degenerate). In the "coupled state" column we give the indexes of states that couple to the given electronic states by vibrations. If the vibration couples to the same states (diagonal term in the electron-vibration Hamiltonian) then the text is written by a normal black ink otherwise it is indicated by red color. We note that more than one state may couple to the given peak in the spectral function. These multiple states are separated by a semicolon in the row. In the column "mode" we provide the indexes of the vibration modes that contribute the most to the electron-vibration coupling for the given peak of the given electronic state. A larger index indicates a larger vibration frequency. More-than-one vibration mode is separated by semicolon in the row. The corresponding symmetry labels and their frequency in meV unit are given in the columns "symmetry" and "frequency", respectively. Finally, we give the contribution of the given pairs of states coupled by the given vibration in the corresponding peak of spectral function (column "residual"). We provide here only the most significant contributions.

We note here that the order of KS-DFT LDA levels and the GW QP energies changes in urotropine, that is, HOMO − 1(LDA)→HOMO − 3(QP), HOMO − 2(LDA)→HOMO − 1(QP), HOMO − 3(LDA)→HOMO − 2(QP), respectively. This occurs due to the varying GW QP corrections on these levels.

## Supplementary Methods

### ***Ab initio*** **calculation of the broadening of the quasi-particle levels**

According to Born-Oppenheimer approximation, the electrons follow the motion of nuclei instantly, giving the possibility to define the potential energy surface (PES). If this surface can be approximated with a multidimensional parabola then the wavefunction of nuclei will be quantum harmonic oscillators. We assume that the Born-Oppenheimer approximation holds both in the neutral and ionized states, and their potential energy surfaces can be calculated by density functional theory (DFT). The potential energy surface of the neutral and ionized states differs. As a consequence, vibration assisted broadening of the quasi-particle levels may appear in the PES where its amplitude for a given quasi-particle state may be calculated as

$$A \sim |\langle \Phi_\mathrm{N} | \Phi_\mathrm{I} \rangle|^2 \,, \qquad (1)$$

where $\Phi_\mathrm{N}$ and $\Phi_\mathrm{I}$ wavefunctions are the many-body harmonic oscillators of the neutral ($N$) and the ionized ($I$) systems, respectively. We apply Huang-Rhys (HR) approximation to calculate the integral in Supplementary Eq. (1) which assumes displaced multidimensional parabolas between the

neutral and ionized systems. In this case the $(3M-6)^2$ dimensional problem reduces to $(3M-6)$ one-dimensional problems where $M$ is the number of atoms in the nanodiamonds with $(3M-6)$ independent harmonic oscillators. For a given harmonic oscillator $\lambda$ the overlap can be calculated as

$$\left\langle \Phi_m^{(\lambda)} | \Phi_n^{(\lambda)} \right\rangle = \int dx \psi_m(x) \psi_n(x - 2d_\lambda) = \int dx \psi_m(x + d_\lambda) \psi_n(x - d_\lambda) = \frac{1}{\sqrt{2^{n+m} n! m!}} \pi^{-\frac{1}{2}} e^{-d^2} \int dx e^{-x^2} \mathrm{H}_m(x - d_\lambda) \mathrm{H}_n(x + d_\lambda), \tag{2}$$

where the $H_n(x)^{(\lambda)}$ is the $n$th Hermite polynomial, and $\phi_m(x)^{(\lambda)}$ is the wavefunction of the dimensionless quantum harmonic oscillator. The displacement $d_\lambda$ of along mode $\lambda$ is related directly to the partial HR factors $S_\lambda$

$$2d_\lambda^2 = S_\lambda = \omega_\lambda q_\lambda^2/(2\hbar), \tag{3}$$

where the corresponding $q_\lambda$ is defined in the Method section of the main text. With the properties of the Hermite polynomials, the overlap $(O_\lambda)$ for the mode $\lambda$ in Supplementary Eq. 2 can be transformed into a finite series,

$$O_\lambda(m \to n) \sim \sum_{k=0}^{k \le n,m} \binom{n}{k} \binom{m}{k} (2d_\lambda)^{n+m-2k} 2^{k-\frac{n+m}{2}} \frac{k!}{\sqrt{n!m!}} (-1)^k, \tag{4}$$

where $m$ and $n$ label the vibration quantum numbers of the neutral and ionized systems, respectively. The summation of $k$ goes up to either $n$ or $m$, whichever is larger. Here we can further simplify this equation by recognizing the fact that the lowest energy of the vibration modes are larger in all the considered diamondoids than the measurement temperature (room temperature). This means that $m = 0$ for each $\lambda$ in practice, that is, the room temperature spectrum should be almost equal to the zero kelvin spectrum. As a consequence, the summation in Supplementary Eq. 4 reduces, and the amplitude of the vibration assisted sidebands, $0 \to n$ can be calculated as

$$A_\lambda(0 \to n) = |O_\lambda(0 \to n)|^2 = \frac{2d_\lambda^{2n}}{n!} = \frac{S_\lambda^n}{n!}, \tag{5}$$

where $n = 0$ gives the amplitude of the pure quasi-particle state and the rest are the vibration assisted bands that broaden quasi-particle peak. It is clear from Supplementary Eq. 5 that the amplitude of the vibration sidebands decays exponentially as a function of the quantum number of $n$ for a given $\lambda$. The amplitude becomes approximately zero at $n = 10$ for any vibration mode $\lambda$ for the considered diamondoids. The partial HR spectral function can be defined as $A_\lambda(\hbar\omega) = \frac{S_\lambda^n}{n!} \delta(\hbar\omega - n\hbar\omega_\lambda)$. The total vibration sideband can be calculated as a convolution ($\ast$)

of $(3M-6)$ partial HR spectral functions,

$$A(\hbar\omega) = \mathop{\ast}_{\lambda=1}^{3M-6} A_\lambda(\hbar\omega) = \mathop{\ast}_{\lambda=1}^{3M-6} \sum_{n=0}^{\infty} \frac{S_\lambda^n}{n!} \delta(\hbar\omega - n\hbar\omega_\lambda) \,. \tag{6}$$

Finally, by defining the total HR factor as $S = \sum_\lambda S_\lambda$ and an average vibration mode as $\overline{\omega} = \sum_\lambda \omega_\lambda S_\lambda / S$ one can approximate a continuous envelope function of $A(\hbar\omega)$ by

$$\frac{S^n}{n!} \delta(\hbar\omega - n\hbar\overline{\omega}) \,, \tag{7}$$

where $n$ can be chosen to be continuous by taking $n! = \Gamma(n+1)$, and evaluate the $\Gamma$ function between $n = [-1..\infty]$. We applied this simplified function to broaden the quasi-particle levels and the corresponding many-body perturbation theory spectral functions.

For the HOMO levels of adamantane, diamantane and urotropine we show the calculated spectrum in detail in Supplementary Figure 1. In the detailed spectrum some peaks can be identified in the photo-emission spectrum (black curve) of adamantane and diamantane as the pure quasi-particle peak and the vibration assisted peaks. The red curve plots the calculated $A(\hbar\omega)$ in Supplementary Eq. 6 which shows a non-symmetrical function with fine structure that originates from vibration modes coupled strongly to the ionization process. In the case of adamantane and diamantane the calculated fine structure is visible for $n = 4$ of their corresponding average vibration modes. Whereas some peaks in PES may be identified as these resonances, nevertheless, we rather focus on the broadening of the spectrum as defined in Supplementary Eq. 7 (blue curve). The overall width of the broadening of the HOMO is ≈1.2 eV in the considered diamondoids.
